# Recent Advancements in Epidural Etanercept for Pain Management in Radiculopathy: A Literature Review

**DOI:** 10.7759/cureus.37672

**Published:** 2023-04-17

**Authors:** Andrew M Joseph, Monica Karas, Ernesto Joubran, Cesar E Jara Silva, Steven Cordova, Mehul Sinha, Abdus Salam, Melissa M Leyva, Jonathan Quinonez, Samir Ruxmohan

**Affiliations:** 1 Department of Osteopathic Medicine, Nova Southeastern University Dr. Kiran C. Patel College of Osteopathic Medicine, Davie, USA; 2 Department of Osteopathic Medicine, Nova Southeastern University Dr. Kiran C. Patel College of Osteopathic Medicine, Fort Lauderdale, USA; 3 Department of Neurology, Larkin Community Hospital, South Miami, USA; 4 College of Medicine, St. Matthew's University School of Medicine, Grand Cayman, CYM; 5 Department of Medicine, International Society for Chronic Illnesses, Vadodara, IND; 6 Department of Surgery, Kasturba Medical College, Mangalore, IND; 7 Department of General Surgery, Khyber Teaching Hospital, Peshawar, PAK; 8 Department of Neurology/Osteopathic Neuromuscular Medicine, Larkin Community Hospital, Miami, USA; 9 Division of Neurocritical Care, UT Southwestern Medical Center, Dallas, USA

**Keywords:** tumor necrosis factor alpha inhibitor, degenerative disc disease, radiculopathy, pain management, epidural etanercept

## Abstract

The most common etiology of low back and neck pain is associated with spinal cord pathologies. Regardless of origin, low back and neck pain are some of the most common causes of disability worldwide. Mechanical compression due to spinal cord diseases, such as degenerative disc disorders, can lead to radiculopathy, which manifests as numbness or tingling and can progress to loss of muscle function. Conservative management, such as physical therapy, has not been proven effective in treating radiculopathy, and surgical treatments have more risks than benefits for most patients. Epidural disease-modifying medications, such as Etanercept, have been recently explored due to their minimal invasiveness and direct effects on inhibiting tumor necrosis factor-α (TNF-α). Therefore, this literature review aims to evaluate epidural Etanercept's effect on radiculopathy caused by degenerative disc diseases. Epidural Etanercept has been shown to improve radiculopathy in patients with lumbar disc degeneration, spinal stenosis, and sciatica. Further research is needed to compare the effectiveness of Etanercept with commonly used treatments such as steroids and analgesia.

## Introduction and background

Low back and neck pain are some of the leading causes of disability in developed countries [1]. Spinal cord diseases are among the most common etiologies leading to neck and low back pain [1]. Mechanical compression of the spinal cord and nerve roots, due to degenerative changes, can lead to radiculopathy, which is defined as numbness or tingling with pain radiation, and can progress to loss of muscle function [2]. Degeneration of the vertebral joints and foramina contributes to the pain in radiculopathy [3], including spinal cord tumors, disc herniations, or spinal stenosis. While conservative, physical therapy and complementary health approaches have not proven effective in the pain management of spinal cord degenerative diseases [4]. Surgical interventions pose significant risks of adverse outcomes and failures [3]. Oral medications and epidural pain management preclude invasive surgeries [2]. Epidural disease-modifying medications, such as Etanercept, have been recently explored due to their minimal invasiveness and direct effects on inhibiting tumor necrosis factor-α (TNF-α) [3]. TNF-α is a cytokine with pro-inflammatory properties that increases the expression of endothelial selectin, leading to acute-phase protein production within the liver that can be seen in pathology such as radiculopathy [3]. As such, inhibiting this cascade could potentially decrease inflammation and pain experienced [3]. Therefore, this literature review aims to evaluate epidural Etanercept's effect on radiculopathy caused by degenerative disc diseases.

Etiologies of radiculopathy

Secondary to degenerative etiologies, mechanical compression can be attributed to spinal cord diseases [5]. Such structural abnormalities lead to some of the most common clinical complaints: neck and back pain [2,4]. In developed countries, it is estimated that the incidence of low back pain and neck pain account for 13% to 30% of all disabilities [5]. Spinal nerves can experience noxious stimuli from different sources, leading to ectopic signals that can be perceived as pain and numbness [6]. Any mechanism irritating spinal nerves can present as radicular pain [6]. Differential diagnoses include trauma, tumors, vascular conditions, infections, and degenerative diseases [6]. The most common etiologies of lumbar radiculopathies are degenerative spine diseases and intervertebral disc lesions [1,4]. As one of the most common problems for a neurological consultation, lumbosacral radiculopathy has an estimated prevalence of around 3% to 5% in adults and ranges equally among males and females [7].

Similarly, radicular symptoms in cervical distributions may appear as pain involving the neck, shoulder, or arm and motor or sensory dysfunction [7]. The mean age of diagnosis is approximately in the fifth decade [6]. Incidence rates per 100,000 people were 107.3 in males and 63.5 in females [6]. The C7 cervical root accounts for approximately 70% of patients with cervical radiculopathy, followed by C6 with 20 percent [7]. The remaining 10 percent is distributed among the C5, C8, and T1 spinal levels [7]. Comparable to the lumbosacral region, the predominant pathological mechanism in the cervical region is compressive radiculopathy, in which spinal degeneration and disc herniation are among the most common [3].

Anatomy, pathophysiology, and clinical features of spinal cord diseases

The vertebral column comprises a detailed and well-functioning organized structure that protects the central nervous system, specifically the spinal cord [8]. Distributed by regions, the spinal column adapts to the body's motions and provides an effective environment for the spinal cord and its divisions [8,9]. Posterior connections join cervical vertebral bodies through the zygapophyseal (facet) joints and the uncovertebral joints, or joints of Luschka, which are the point of contact through the vertebral body lateral surface [1,9,10]. 

Deviation of the zygapophyseal joint can cause pain through the dorsal ramus of the exiting spinal nerve and bony overgrowth from the uncovertebral joints [1,2]. Pathological or physiological degeneration of the vertebral joints and foramina contributes to the perceived pain in radiculopathy [2,3]. Spondylosis decreases the height of the spinal disc, increasing stress at the joint of Luschka, facets, and vertebral foramina, resulting in hypertrophy of the ligaments and osteophyte formation [1,3]. Furthermore, disc herniation can also cause mechanical compression in radiculopathy [1,2,8]. Degeneration of ligamentous fibers and intervertebral pressure can tear the annulus, leading the nucleus pulposus to prolapse and press on a nerve root [2,8]. The combination of mechanical force and subsequent inflammatory response allows neurological symptoms to appear [8]. The clinical presentation varies according to the nerve root, ganglion, or spinal nerve affected [1,3]. Acute presentation occurs mainly from herniations, while the indolent course is in degenerative causes [10]. Symptoms can appear across various individual or combined abnormalities in pain, numbness, motor weakness, or reflexes [2].

Current approaches to pain management

Epidural therapies are injected into the epidural space, the area between the vertebral wall and the dura mater which contains blood vessels, adipose tissue, and spinal nerve roots, that target neuroinflammatory pain, including cervical transforaminal fluoroscopically guided epidural steroid injection (CTFESI), to treat radicular pain [11]. Parasagittal interlaminar (PIL) epidural steroid injection (ESI) has demonstrated increased pain relief with more significant improvement in functionality postprocedure [11,12]. Current approaches of injection include transforaminal and midline interlaminar. Each of these has demonstrated an increased risk of paraplegia, paralysis, and intradiscal injection of the medication [11]. Because PIL has shown a more efficacious distribution of medication compared to the other approaches and possesses a lower risk of adverse effects, PIL may be safer and more effective than transforaminal or midline interlaminar injections for chronic low back pain [11,12,13].

Pain management for neuropathic and neuroinflammatory pain includes epidural analgesia with or without steroids. Epidural analgesia for spinal fusion postoperative pain 24 hours after surgery revealed a pain reduction of 17.2 points on the visual analog scale (VAS) in the meta-analysis of three trials [14]. The VAS utilized is a tool to quantify pain intensity [14]. One meta-analysis showed no significant difference in lumbar disc herniation and spinal stenosis in patients who received lidocaine alone versus lidocaine with steroids for one to two years [14,15]. Epidural steroids with anesthetic resulted in significantly more Numeric Rating Scale (NRS-11) and Oswestry Disability Index (ODI) improvement than epidural anesthetics alone after one year for Degenerative Disc Disease (DDD). More specifically, ODI improvement has been demonstrated for two years in patients with lumbar DDD treated with epidural steroids and anesthetics [15,16,17,18].

Intrathecal management for neuropathic pain has also been explored in research studies. Briefly, the intrathecal space is found between the pia mater and the arachnoid mater and houses the cerebrospinal fluid (CSF) that bathes the spinal cord [19,20,21]. Ziconotide intrathecal drug therapy (IDT) has shown promise in a small trial (*n* = 14) to have a subjective improvement with neuropathic pain [18]. Opioids, with or without bupivacaine, delivered intrathecally have helped manage pain in another small trial (*n *= 16) [19]. More extensive trials on intrathecal pump delivery of analgesics like ziconotide are needed to see if this can effectively manage failed back surgery syndrome [20,21].

While conservative approaches and widely available, physical therapy and complementary health approaches have not proven effective in the pain management of spinal cord degenerative diseases [4]. Surgical interventions, such as discectomies, arthroplasties, laminectomies, laminotomies, and spinal fusions, have been well-established treatment options [22]. Oral medication and epidural pain management complement or preclude these invasive procedures. With respect to other surgical interventions, epidural etanercept is minimally invasive and provides direct physiologic effects such as anti-inflammation which can help curb radiculopathy symptoms caused by degenerative disc diseases [22]. As such, this literature review aims to evaluate epidural etanercept efficacy on radiculopathy secondary to degenerative disc diseases.

## Review

Methods and results

A computerized search utilizing Google Scholar, PubMed, OVID Medline, Embase, Cochrane, and Web of Science was carried out to identify published literature on epidural Etanercept for pain management in radiculopathy. Before moving forward in the review, a set of inclusion and exclusion criteria were generated. Articles included must have been published between 2015 and 2023, written in English, and include the keywords "epidural etanercept" and "radiculopathy." Using the aforementioned search criteria, a total of 128 articles were identified. Thirty-one duplicates were removed from these articles, bringing the total to 97 articles. In reviewing the title and abstract of the remaining articles, 68 of them were removed due to not containing "epidural etanercept" and "radiculopathy." Two reviewers reviewed the remaining 29 articles to assess if it was pertinent and in the scope of this review. A final list of eight articles discussed the utilization of epidural Etanercept for pain management in radiculopathy. Table 1 reports the pertinent characteristics of the articles included in this review. 

**Table 1:** Data extraction table. VAS, visual analog scale; ODI:, Oswestry Back-Related Disability Index; IL, interleukin; TGF, transforming growth factor; TNF, tumor necrosis factor; CCI, chronic constriction injuries; PIVD, prolapse intervertebral disc; IV, intravenously; N/A, not available

Reference	Purpose	Study type	Study description	Key findings and limitations
Dagar et al. (2017) [23]	To assess leg pain and back pain, secondary to sciatica, after receiving transforaminal epidural Etanercept	Prospective Study	Injections (2 mg) of Etanercept was administered two weeks apart to 33 patients. Leg pain and back pain were measured using the VAS for primary outcomes. The modified ODI was used for secondary outcomes.	There was a statistically significant reduction in VAS for leg pain, back pain, and ODI in 31 of the 33 patients (*P* < 0.001). No adverse events were reported.
Hung et al. (2017) [24]	To identify the role of cytokines in the neuropathic pain cascade	Literature Review	Pro-inflammatory IL-1B, IL-6, IL-17, and TNF-α cytokines, along with anti-inflammatory IL-4, IL-10, and TGF-beta cytokines, were assessed for the location of the action, types of neuropathic pain, and current medications that target them.	Neuropathic pain is associated with elevated IL-1β, IL-6, IL-17, and TNF-α. Therapies for neuropathic pain that modify the levels of cytokines have potential. Further studies should investigate how efficacious these medications are for neuropathic pain.
Wei et al. (2020) [25]	To identify the efficacy of TNF-α epidural injections for patients with radicular pain secondary to lumbar spinal stenosis lasting more than six weeks	Randomized Controlled Trial	A total of 90 patients were randomly assigned to groups receiving epidural TNF-α inhibitor, steroid, or lidocaine only. The patients were assessed with VAS and ODI for function.	Pain relief and overall movement function improved significantly with the epidural TNF-α inhibitor when compared to both the steroid and lidocaine groups (*P* < 0.05). No statistical difference was found between the steroid and lidocaine groups in pain relief and overall movement function (*P* > 0.05).
Gerard et al. (2015) [26]	To evaluate the role of TNF-α in the expression of hyperalgesia and allodynia in rats with CCI	Animal Experiment	A total of 42 male Sprague-Dawley rats were included in this study. Rats received stereotaxic surgery and then were split evenly into two groups: a sham group and a CCI group.	Increased TNF-α in the hippocampus was necessary for hyperalgesia and allodynia. Decreasing TNF-α through anti-TNF-α inhibitors results in the inhibition of neuropathic pain, resulting in efficacious chronic pain therapy.
Amin et al. (2017) [27]	To evaluate the current evidence on treatments for lumbar disc herniation	Narrative Review	N/A	Epidural Etanercept showed significant improvement in leg and back pain in a placebo-controlled randomized trial. However, this trial mentioned in the review did not evaluate dose-dependent responses or form a comparison group with local steroid injections.
Johnson et al. (2015) [28]	To evaluate the role of TNF-α and IL-1β in disc degeneration and low back pain and discuss new therapeutic management	Narrative Review	N/A	TNF-α and IL-1β have been shown to disrupt the extracellular matrix, resulting in disc degeneration. Their presence also activates an inflammatory response that results in pain and further disc degeneration. TNF-α inhibitors administered during surgery have been shown to stop macrophage infiltration. Several clinical trials explored in this review showed improvement in pain and disability through epidural Etanercept administered to patients with lumbar disc herniation, lumbar spinal stenosis, subacute lumbosacral radiculopathy, and severe sciatica. However, a multicenter study revealed decreased efficacy compared to epidural steroids in patients with lumbosacral radiculopathy.
Singh et al. (2020) [29]	To discuss the management and etiology of lumbar PIVD	Narrative Review	N/A	PIVD patients had better outcomes and showed improvement in pain when administered transforaminal epidural anti-TNF-α inhibitors. The decubitus position was found to be more effective for epidural administration.
Beyaz et al. (2017) [30]	To compare the effectiveness of TNF-α antagonists, infliximab and Etanercept, administered IV or epidural in lumbar spine diseases	Animal Experiment	A total of 24 Sprague-Dawley male rats with discopathy leading to radiculopathy-related allodynia were included in this study. Rats were assigned to four different groups. Group 1 rats were treated only surgically. Rats assigned to group 2 received 1 mL of epidural saline. Group 3 rats received 10 mg/kg of infliximab IV through the coccygeal vein. Group 4 rats received an epidural of 25 mg of Etanercept. Left leg pull responses were used to measure recovery time in all four groups on the baseline and then on days 7, 14, 21, and 28.	In group 1, all responses were similar to the baseline. Both groups 2 and 3 had the same left leg responses on days 21 and 28 compared to baseline, indicating recovery time that started on day 21. Therefore, no significant difference in recovery time was noted between the rats who received IV saline versus those who received IV infliximab. Group 4 leg pull responses were similar to baseline on days 14, 21, and 28, indicating that recovery time started earlier than day 14. Rats who received epidural Etanercept recovered earlier than rats who received IV infliximab and epidural saline. To confirm, a single dose of epidural Etanercept was administered immediately after surgery, which revealed that allodynia ended earlier, indicating that epidural Etanercept is more effective than systemic anti-TNF-α infliximab.

Discussion

**TNF-** *α* ***a*nd Radiculopathy**

Radiculopathy can be defined as a sharp shooting pain radiating from one body part to another of which a common example seen in the primary care setting is sciatica caused by a lumbar disc herniation [23]. Sciatica is a pathology that involves a sharp or burning sensation that is often attributed to mechanical sciatic nerve compression due to disc herniation or prolapse [23]. However, later, research revealed that some patients having large disc herniations had no radicular symptoms, whereas others with only mild disc bulges had severe radiculopathy [23]. The intervertebral disc comprises three components: cartilaginous endplates, outer annulus fibrosus, and inner nucleus pulposus [23]. There is no blood supply to the nucleus pulposus, leading to immune privilege [23]. A disc herniation will result in the rupturing of the annulus fibrosis, which leads to the exposure of the nucleus pulposus [23]. This exposure results in an autoimmune response with acute inflammation [23]. The mechanisms for inflammatory response following disc herniation are as follows: First, releasing several cytokines, such as TNF-α and IL-1, attracts neutrophils and macrophages to the injury site. Some studies have demonstrated that disc cells produced CCL5/RANTES, a cytokine attracting eosinophils and macrophages, after treatment with TNF-α and IL-1 [28]. The expression of CCL5/RANTES is associated with discogenic pain and the severity of degeneration [28].

Second, increased levels of TNF-α from nucleus pulposus samples have been found in adults with painful disc herniation compared to healthy controls [28]. Herniated disc cells produce leukocytes that attract and produce IL-1 and TNF-α [28]. It has been demonstrated that nucleus pulposus tissue expresses both the TNF receptors TNFR1 and TNFR2 [28]. Expression of TNFR1 has been associated with an increased degree of pain [28]. A higher level of expression of TNFR1 is linked with severe forms of low back pain [28]. TNF-α and IL-1β levels have been positively correlated with the degree of severity of disc degeneration [28]. More evidently, the expression of pro-inflammatory cytokines has been found to correlate with the severity of disc degeneration and play a major role in its pathogenesis [28]. Thus, TNF-α has been shown to play a major role in disc degeneration-related neuropathic pain [28]. TNF-α is also necessary for expressing hyperalgesia and allodynia in a randomized controlled trial on 42 Sprague-Dawley rats [26].

Another mechanism of action of TNF-α in disc herniation is through induction of certain catabolic enzymes A-Disintegrin and Metalloproteinase with Thrombospondin Motifs (ADAMTS) 4 and 5, and matrix metalloproteinases (MMPs) [28]. A concurrent decrease in the expression of anabolic extracellular proteins aggrecan and type II collagen is also implicated in the mechanism of disc herniation [28]. The heparan-sulfate proteoglycan syndecan-4 pathway and NF-kB signaling pathway have also been found to play a major role in the mechanism of action of TNF-α and IL-1 [28]. TNF-α has been shown to activate the Wnt/β-catenin pathway, increasing the expression of MMP [28]. MMPs have a major role in causing the degeneration of intervertebral discs, thus leading to disc herniation and radiculopathy.

In addition to the aforementioned mechanisms, annulus fibrosus exposed to both IL-1 and TNF-α resulted in a significant decrease in growth differentiation factor 5 (GDF5) levels, which was found to play an important anabolic role in disc development [28]. Another signal cascade that was noted to be activated in patients with disc degeneration compared to those who had nondegenerated discs was the Notch pathway [28]. While there is no literature on the role of its activation with disc degeneration, this pathway has been shown to play a role in malignant transformation [28]. It was found that both NOTCH1 and NOTCH2, both of which are products of this signal pathway, and their associated receptors were increasingly expressed when the nucleus pulpous was exposed to TNF-α and IL-1 [28]. Thus, TNF-α regulation of the Notch pathway has been shown to play a major role in cellular growth and differentiation in disc degeneration [28].

Last but not least, other studies have also shown that TNF-α plays a major role in the water channels of the nucleus pulposus. Some studies show that TNF-α exposure to the nucleus pulposus resulted in decreased hydraulic permeability and cell radius [28]. This may be due to the decreased number of Aquaporin 1 receptors, a type of water channel [28]. The decrease in Aquaporin 1 receptors has been associated with increased severity of disc degeneration [28].

Pharmacokinetics and Pharmacodynamics of Epidural Etanercept

Etanercept, a TNF inhibitor, is now approved by the U.S. Food and Drug Administration (FDA) for treating painful disorders of the extremities and axial spines such as psoriatic arthritis and ankylosing spondylitis [24]. While current literature describes the pharmacodynamics and pharmacokinetics of subcutaneous etanercept, there continues to be ambiguity on the half-life, peak, and duration of action of the epidural form [24].

TNF antagonists have shown inconsistent outcomes in clinical studies. In a dose-response and placebo-controlled study, 14 out of 18 radiculopathy patients received 2, 4, or 6 mg of transforaminal epidural Etanercept [24]. When pain levels were measured at one and six months, long-term leg pain relief was reported in the Etanercept group compared to one out of six patients in the control group who received saline [24]. The trial's shortcomings were limited sample size and Etanercept's uncertain therapeutic dosage range [24]. Also, patients who did not respond in both the control and Etanercept groups were not tracked for more than one month, as per study protocol [24].

Moreover, Amin et al. demonstrated in a placebo-controlled trial that transforaminal Etanercept significantly improved pain scores through three to six months [27]. However, Etanercept did not demonstrate a dose-dependent response, and no group was used as a reference for corticosteroid injection, raising some questions about the drug's pharmacodynamic properties [27]. On the other hand, a separate randomized controlled trial of 80 lumbar spinal stenosis patients discovered that the safe and efficient epidural injection of 10 mg of Etanercept reduced pain better than dexamethasone, demonstrating a dose-dependent pharmacodynamic effect [24].

During long-term follow-up, the efficacy of lumbar epidural injections varied from 20% to 95% [29]. Transforaminal injections obtained better results than caudal or inter-laminar methods [29]. The patient's position was found to potentially impact the pharmacokinetics and pharmacodynamics of the medication during the injection [29]. The lateral decubitus position resulted in superior results at six and 12 months when compared to prone positioning [29]. Etanercept is also often more effective when administered locally than systemically [23].

While it is known that Etanercept blocks the activity of TNF-α, the exact role of TNF-α in radiculopathy pain is still unclear [24,25]. The cost-effectiveness of Etanercept in radiculopathy pain is also questionable, as it is an expensive drug that may not be affordable or accessible for many patients [23]. In addition, Etanercept may have different effects on different types of radiculopathy pain, making it less effective for some patients [26].

Etanercept may also have adverse effects, including injection site reactions, infections, allergic reactions, and an increased risk of malignancies. These risks may outweigh the benefits of Etanercept in mild or self-limited pain [23,24]. Moreover, Etanercept may not be effective for all patients with radiculopathy pain, as the response to Etanercept may depend on factors such as the duration, severity, and location of pain, the presence of inflammation or infection, and the genetic profile of the patient [23,24,25].

Finally, Etanercept may interact with other medications or supplements the patient takes, increasing the risk of bleeding or infection when combined with anticoagulants or immunosuppressants [23]. It may also be unsuitable for pregnant or breastfeeding women, children, or elderly patients due to potential risks of fetal malformations, growth retardation, or immune system impairment [23].

Epidural Etanercept Utilization in Radiculopathy 

Radiculopathy pain is a debilitating condition that can severely affect a patient's well-being. Etanercept is a biological drug used to treat radiculopathy pain, but its limitations must be considered. Research studies on the effect of epidural Etanercept on radiculopathy have been increasingly growing in recent years. This literature review aims to discuss epidural Etanercept's efficacy on radiculopathy through prospective studies, reviews, and randomized controlled trials published in or after 2015. 

Randomized human clinical trials and prospective studies also tested the hypothesis that TNF-α inhibition improved radiculopathy by measuring pain on a visual analog scale [23,25,26,27,28,29]. Dagar et al. administered two doses of epidural Etanercept two weeks apart to 33 sciatica patients presenting with leg and back pains [17]. Leg pain, back pain, and ODI were significantly reduced in all 33 patients, with no reported adverse effects [23]. A randomized control trial then compared the efficacy of Etanercept to steroids and analgesia [25]. In this clinical trial, 90 patients with lumbar spinal stenosis who presented with radicular pain for more than six weeks were randomly assigned to receive either epidural anti-TNF-α, steroid, or lidocaine only [25]. Results were measured using the visual analog scale and ODI [25]. Rats treated with epidural TNF-α inhibitors showed significantly improved pain relief and movement function compared to both steroid and lidocaine treatment groups [25]. Steroid and lidocaine had no statistical difference, indicating that they were equally efficacious in pain relief and movement function [25]. 

Finally, narrative reviews also supported the randomized clinical trials and animal experiments. A review of the current treatments of lumbar disc herniation revealed that epidural Etanercept significantly improved leg and back pain in a placebo-controlled randomized trial [27]. Another review on disc degenerative disease revealed that TNF-α inhibitors administered during surgery had been shown to stop macrophage infiltration [28]. Several clinical trials explored in this review demonstrated that epidural Etanercept improved pain and disability in patients with subacute lumbosacral radiculopathy, lumbar disc herniation, lumbar spinal stenosis, and sciatica [28]. One multicenter study mentioned in this review demonstrated decreased efficacy of epidural Etanercept when compared to epidural steroids in patients with lumbosacral radiculopathy; however, no clinical trial has supported these conclusions [28]. Lastly, another review on patients with lumbar prolapse intervertebral disc (PIVD) had significant pain relief when treated with transforaminal epidural anti-TNF-α inhibitors [30]. Specifically, this review demonstrated that the lateral decubitus position was most efficacious for epidural administration when comparing results six months postadministration [30]. 

Limitations of this study

This review only assessed articles written in English due to a lack of translational resources. In addition, different studies utilized various protocols and reported variables and results. This made it difficult to determine the best way to utilize them in clinical practice. Another limitation is the lack of long-term human studies on the efficacy and safety of Etanercept for pain in radiculopathy. Most studies have followed patients for only a few weeks or months, making it unclear if Etanercept can relieve pain or prevent further nerve damage or disability. Therefore, the inclusion of animal studies in this review was deemed necessary. A comparison of other epidural TNF-α inhibitors was not completed in this review. As such, no conclusions can be drawn regarding how Etanercept compares to other TNF-α inhibitors.

Future recommendations

Etanercept is a promising potential treatment for pain in radiculopathy, but there remains room for improvement. There are several potential areas for improvement in using Etanercept for radiculopathy pain. These include conducting more high-quality studies to compare Etanercept with other treatments and evaluating the optimal dose, frequency, and duration of etanercept treatment for different types of radiculopathies. Also, more studies are needed to assess the long-term outcomes and adverse effects of Etanercept treatment. Studies are also needed to explore the mechanisms of action and potential biomarkers of Etanercept therapy and investigate the use of Etanercept for other types of radiculopathies. By focusing on these areas, healthcare professionals may be able to improve treatment options and outcomes for patients with radiculopathy pain.

## Conclusions

Radiculopathy pain is a debilitating condition that can radically affect a patient's well-being. Etanercept is a biological drug used to treat pain due to radiculopathy, but its limitations must be considered. Research studies on the effect of epidural Etanercept on radiculopathy have been increasingly growing in recent years. Epidural Etanercept has been shown to improve radiculopathy in patients with lumbar spinal stenosis, lumbar disc degeneration, and sciatica. Further research is needed to compare the effectiveness of Etanercept with commonly used treatments such as steroids and analgesia. 
